# Influence of Commercial Insurance Purchase on the Health Status of Chinese Residents

**DOI:** 10.3389/fpubh.2021.752530

**Published:** 2021-09-16

**Authors:** Bao-Chang Xu, Xiu-Juan Li, Meng-Yao Gao

**Affiliations:** School of Economics, Qingdao University, Qingdao, China

**Keywords:** commercial insurance, health status, China, ordered logit, CGSS2015

## Abstract

Under the context of rapid economic and social development, and growing demands for a better life, Chinese residents have been increasingly concerned with their health status and issues. In this study, the internal relations between the purchase of commercial insurance by residents and their health status are analyzed and studied with a polytomous logit model based on the data of Chinese General Social Survey (CGSS) in 2015. According to the research result, purchase of commercial insurance significantly improved the health status of residents, with an improving effect for rural residents apparently better than that among urban residents. In addition, purchase of commercial insurance can promote the health status of residents by increasing their household income. This research will provide an effective reference for the innovative development and medical reform of the commercial insurance of China in the future, which is theoretically and practically significant to the implementation of the Healthy China Strategy.

## Introduction

With the rapid development of the economy and society, the pursuit of health among Chinese residents keeps rising, which is mainly shown in their growing demands for social security and increasing expenditure of commercial insurance. Since the reform and opening, Chinese government, social capital, and individuals have gradually increased investment on health. Relevant national statistics show that, from 1978 to 2019, government expenditure on health rose from 3.544 billion yuan to 1.8 trillion yuan, and total expenditure on health rose from 11.021 billion yuan to 6.58 trillion yuan. Accordingly, the total expenditure on health as a percentage of GDP rose from 3 to 6.64%, which was more than doubled (1), among which, the out-of-pocket health payments of an individual also rose from 2.252 billion yuan to 1.87 trillion yuan. Meanwhile, on the basis of implementing the “Healthy China Strategy”, General Secretary Xi Jinping emphasized that “The people's health is an important symbol of national strength and prosperity”. A series of policies and statistics have fully proven that the Party and the country have given top priority to the health of the people.

Social security is the most significant factor in the health status of urban and rural residents. After 70 years of development and change, China has set up the world's largest social security system, and has formed an institutional model and theoretical system of social security more suitable for China's condition, in light of the current situation of Chinese social development, bringing about huge positive changes to the lives of people. Commercial health insurance has played a vital part in the growing demands of residents for personalized security. In 2019, China's premium income of health insurance was 706.6 billion yuan. Statistics show that this income will exceed 1 trillion yuan in 2023 (2). The “Outline of the 14th Five-Year Plan for National Economic and Social Development and the Long-Range Objectives Through the Year 2035 of the PRC,” issued in 2021, has clearly pointed out the necessity to deepen the reform of insurance companies and improve the guarantee capability of commercial insurance. In the meantime, the rapidly developing internet technology has made mobile intelligent terminals more popular. As a result, the change in consumer group of commercial insurance and the strengthened awareness of insurance will have more obvious influence on the health status of Chinese residents.

Therefore, considering the significance of commercial insurance in promoting medical reform and improving the health of residents, this study will explore the influence of commercial insurance purchase on health status of Chinese residents, and study its inherent mechanism.

The rest of the paper is arranged as follows: the first part is the literature review, the second part is the data source and variable selection, the third part is the model selection and empirical results, the fourth part is further discussion, and the fifth part is the conclusion and inspiration.

## Literature Review and Research Hypothesis

Regarding the research of influence factors on health status of residents, most scholars believed that factors, including family structure, social security, income level, access to health information, and preferences for investment in health services, would all have influences on the health status of residents. Based on the data of Chinese General Social Survey (CGSS) in 2008, Sun and Li drew the conclusion that the self-rated health status of residents had significant correlativity with variables of the subjective and objective dimensions, and variables at individual level, such as age could well explain the difference of the self-rated health status of residents (3). Regarding the research of income influencing the health status of residents, Gu and Xiang discovered that the health status of residents would be improved as their income increased, and income increase had greater influence on low income group than high income group (4). During their research on the health status of rural residents, Tong and Luan discovered that rural residents with higher education background and stronger sense of happiness had better health status (5). After tracking the living quality index of residents in 35 cities around the country, Lang et al. discovered that residents with stable living quality index were healthier than expected (6).

Regarding the relationship between insurance and residents, Levy et al. believed that it was necessary to review the relationship between medical insurance coverage and the health status of residents through extensive random experiment, but they did not get ideal experiment results (7, 8). Denise Doiron et al. used relevant data from Australia to study the relationship between ex-ante risk and private medical insurance (9–11). According to the research, there was a significant positive correlation between self-rated health status and private medical insurance (12, 13). In order to further study the relationship between self-rated health status of the elderly and insurance, Polsky et al. took whether the elderly participated in health insurance as the major variable (14, 15). According to the research, after the elderly were covered by the health insurance plan, it was possible to improve their self-rated health status (16). However, according to the research by Card et al., whether the elderly participated, the health insurance would not greatly affect their health status (17). Therefore, there was no consistent conclusion as to the relationship between commercial insurance and the health status of residents.

In the academic world, there were also many studies on the internal influencing mechanism between social security and the health status of residents. Based on the data of Chinese Household Income Project (CHIP) in 2014, Su discovered that appropriate increase in social security benefits could effectively improve the health status of the elderly (18). Based on the data of CGSS in 2013, Li and Zong analyzed the relationship between the expenditure of government in social security and the health status of residents (2). The research result showed that increasing the expenditure in social security could effectively improve the urban and rural health status of residents (19). Sun and Wang used ordered Logistic-ISM model to analyze their structures and relations (1). The research showed that the satisfaction degree of social security among urban residents would increase with the improvement of their health status.

However, there is still the problem of health inequality caused by factors such as regional and wealth disparities of the social security system of China (20, 21). As a result, social security of China cannot fully cover all of the problems of the health status of residents. As the strong complement of social security system, commercial insurance has had considerable influence on health status of urban and rural residents. The research by Liu and Wang and Li et al. showed that enhancing social medical care could significantly increase the demands of residents on commercial health insurance (22, 23). In the research by Gill et al. and Li et al., it was discovered that participating in commercial health insurance could significantly improve the health status of residents (23, 24).

In conclusion, scholars at home and abroad focused on analyzing the impact of individual and family factors on the health of residents, but there were few studies on the analysis of impact of commercial insurance on the health status of urban and rural residents. Therefore, based on the data of CGSS in 2015, this paper analyzed the internal relationship between commercial insurance and self-rated health status of urban and rural residents, so as to further implement the Healthy China Strategy effectively, providing useful reference for promoting the stable development of the commercial insurance of China.

## Data Sources and Specification of Variables

### Data Sources

This article uses data from CGSS in 2015. In this survey, data were comprehensively and systematically collected from many dimensions such as society, family, and individual, providing nationwide and authoritative source of data samples for conducting scientific research and solving practical problems. There were a total of 10,968 valid questionnaires completed in 2015 CGSS. Eight thousand six hundred seventy-five (8,675) valid samples were obtained after data filtering and processing.

### Variables Selection

#### The Explained Variable

The explained variable used in this paper is the health status of residents. There are many forms of measurement standards of health, including physical health and mental health. The SF-36 measurement table is used internationally, but its measurement method cannot control the accuracy in answering questions, and the measurement is quite complicated, so it is not commonly applied. The simplified SF-8 measurement table has high cost, so it is not widely used by domestic scholars. Xiang believed that the self-rated health status had high credibility, and Gu and Xiang also believed that the physical health status and self-rated health status had high levels of consistency (4, 7). Therefore, this paper measures the health status of residents from a subjective point of view, and adopts the residents' self-rated health index for measurement, using numbers from 1 to 5 to show the increasing degree of residents' self-rated health status.

#### The Core Explained Variable

The core explained variable used in this paper is whether to purchase commercial insurance, wherein commercial insurance includes commercial endowment insurance and commercial medical insurance. Based on the data of CGSS in 2015 (CGSS2015), there were 10,968 samples, among which 7,908 people did not purchase commercial medical insurance, while 767 did. Eight thousand one hundred fifty-nine (8,159) people did not purchase commercial endowment insurance, while 516 did.

#### The Control Variables

The control variables of this paper are selected from three dimensions including society, family, and individual. The detailed control variables are as shown in Table 1.

**Table 1:** Variable definition and assignment.

**Variable**	**Variable assignment**
Health	From 1 to 5, get better
Binsurance	Yes = 1, No = 0
Age	Age (years)
Age_2	Age squared
Gender	Male = 1, Female = 0
Edu	Yes = 1, No = 0
Marriage	Yes = 1, No = 0
Household	Town = 1, Country = 0
Ln_fincome	Take the logarithm of last year's household income
Neighbor	From 1 to 7, frequency of interaction goes down
Familysize	Number of people living together currently
Sinsurance	Yes = 1, No = 0
Invest	Yes = 1, No = 0

The explained variable is the health status of residents, which is a multi-categorical variable, from 1 to 5 indicating that the health status is getting better and better. As seen from Table 2, the average value of residents' self-rated health status is 3.64, meaning that residents have generally good evaluation of their health status, but further improvement is still needed. The average value of participating in commercial insurance is 0.1, meaning that most of the people did not participate in commercial insurance, and the awareness and sense of participation in commercial insurance of domestic residents need to be improved.

**Table 2:** Descriptive statistics of variables.

**Variable**	**Observation**	**Mean**	**Standard deviation**	**Minimum**	**Maximum**
Health	8,675	3.64	1.05	1	5
Binsurance	8,675	0.10	0.30	0	1
Age	8,675	50.01	16.33	19	85
Age_2	8,675	2,767.26	1668.38	361	7,225
Gender	8,675	0.48	0.50	0	1
Edu	8,675	0.34	0.47	0	1
Marriage	8,675	0.90	0.30	0	1
Household	8,675	0.42	0.49	0	1
Ln_fincome	8,675	10.34	1.76	0	13.12
Neighbor	8,675	3.46	2.02	1	7
Familysize	8,675	2.88	1.36	1	7
Sinsurance	8,675	0.94	0.24	0	1
Invest	8,675	0.91	0.28	0	1

## Model Selection and Empirical Result

### Model Selection

Since the explained variable is polytomous variable, in the data of CGSS in 2015, residents' self-rated health status belongs to the ordered discrete type, wherein the health level is divided into five grades, being very unhealthy, relatively unhealthy, average, relatively healthy, and very healthy, using the numbers from 1 to 5 showing the increasingly better status. Therefore, this article uses ordinary linear squares (OLS) multiple regression model, orderedmultivariate logit model and tobit regression model for empirical research and analysis.

The empirical regression model is set in the paper as below:


(1) $$\begin{array}{l} \log(p_{k})=\theta_{k}+\beta_{1}binsurance+\beta_{2}age+\beta_{3}age\_2\\ \;+\beta_{4}gender+\beta_{5}edu+\beta_{6}marriage+\beta_{7}household\\ \;+\beta_{8}\ln\_fincome+\beta_{9}neighbor+\beta_{10}familysize\\ \;+\beta_{11}\sin surance+\beta_{12}invest+\mu \end{array}$$

Where *p*_*k*_ refers to the probability when residents' self-rated health level y = k, and θ_*k*_ is the intercede when residents' self-rated health level y = *k* (*k* = 1, 2, 3, 4, 5).

If the dependent variable is limited, tobit regression model can be adopted, using the concept of maximum likelihood to conduct empirical survey on the relationship between independent variable and dependent variable. The tobit regression model is built based on hypothesis as below:


(2) $$\begin{array}{l} y=\beta_{0}+\beta_{1}binsurance+\beta_{2}age+\beta_{3}age\_2+\beta_{4}gender\\ \;+\beta_{5}edu+\beta_{6}marriage+\beta_{7}household+\beta_{8}\ln\_fincome\\ \;+\beta_{9}neighbor+\beta_{10}familysize+\beta_{11}\sin surance\\ \;+\beta_{12}invest+\mu \end{array}$$

where y refers to dependent variable, i.e., the residents' self-rated health status, β_0_ refers to constant term, β_1_ refers to the coefficient of core explained variable, β_*i*_ refers to the coefficient of control variable (*i* = 2, 3,., 12), and μ refers to error term of regression.

### Empirical Result

This study investigated the influence of commercial insurance purchase on health status of urban and rural residents. First, regression of full sample data is conducted based on OLS regression model, and then logit regression and tobit regression are conducted and compared. The regression result is shown in Table 3.

**Table 3:** Benchmark regression.

**Variable**	**OLS**		**Logit**		**Tobit**	
	**(1)**	**(2)**	**(3)**	**(4)**	**(5)**	**(6)**
Binsurance	0.3236*** (0.0373)	0.0852** (0.0358)	0.5344*** (0.0645)	0.1796*** (0.0690)	0.3236*** (0.0373)	0.0852** (0.0358)
Age		−0.0487*** (0.0042)		−0.1008*** (0.0083)		−0.0487*** (0.0042)
Age_2		0.0003*** (0.0000)		0.0006*** (0.0001)		0.0003*** (0.0000)
Gender		0.1702*** (0.0208)		0.3332*** (0.0402)		0.1702*** (0.0208)
Edu		0.1361*** (0.0272)		0.2185*** (0.0522)		0.1361*** (0.0271)
Marriage		0.2014*** (0.0439)		0.3500*** (0.0850)		0.2014*** (0.0439)
Household		0.0723*** (0.0248)		0.1300*** (0.0478)		0.0723*** (0.0248)
Ln_fincome		0.0714*** (0.0064)		0.1303*** (0.0129)		0.0714*** (0.0064)
Neighbor		−0.0322*** (0.0054)		−0.0610*** (0.0105)		−0.0322*** (0.0054)
Familysize		−0.0048 (0.0082)		−0.0088 (0.0158)		−0.0048 (0.0081)
Sinsurance		−0.0137 (0.0426)		−0.0221 (0.0831)		−0.0137 (0.0426)
Invest		0.0796** (0.0384)		0.1769** (0.0730)		0.0796** (0.0384)
N	8,675	8,675	8,675	8,675	8,675	8,675
*R* ^2^	0.0086	0.1713	0.0028	0.0663	0.0029	0.0640

The *** and ** in the table respectively show the significance under the significance level of 1% and 5%, and the robust standard errors are shown in the brackets.

As seen from the models (1), (3), and (5) from Table 3, the coefficient obtained by regression when only adding core explained variable is significantly positive, meaning that participating in commercial insurance has positive influence on the health status of residents, that is to say, participating in commercial insurance can significantly improve the health level of residents. In the models (2), (4), and (6), a series of control variables at individual, family, and social level are added, and the obtained regression coefficient of core explained variables is still significantly positive, which again means that the purchase of commercial insurance can improve the health level of residents.

As seen from the regression result of age variable, the regression results have all passed the significance testing, which means that as the age grows, the body function will decline and health status will deteriorate. Also, as seen from the square regression result of age, self-rated health status of residents and age square have the U-shaped relationship. From childhood to the prime of life, self-rated health status of residents is rising, while it is descending from the prime of life to old age. The variable regression of marital status has also passed significance testing, meaning that married people pay more attention to health status. The regression of household registration status has significant result, meaning that urban residents have higher evaluation of health status than rural residents, which also explains that, generally, the high income group, namely the urban residents, pay more attention to health status than the low income group, namely the rural residents, intentionally improving and enhancing their health status. The increase of household income can significantly improve the health level of residents. The reason is that when a family has higher income, the residents will have better economic condition to enhance and improve their health status. The regression result of neighborhood relationship shows that better neighborhood relationship will put the residents in a better mood, so their health levels will be higher.

### Robustness Test

When the parameters change, in order to investigate whether the explanation of evaluation results by evaluation methods and indicators is stable, it is necessary to conduct robustness test. This study conducts robustness test using the methods of changing database and time-phased regression test.

#### Changing Database

This paper uses the data of China Family Panel Studies (CFPS) in 2018 to conduct robustness test. When choosing variables, the variable of whether to be a party member replaces the variable of investment activities in the control variables, and the variable of whether to participate in medical insurance replaces the variable of whether to participate in basic medical or endowment insurance, while other corresponding variables remain the same.

As can be seen from the logit regression result (Table 4), regression results of commercial insurance on health status of urban and rural residents all passed the significance testing, which means that the regression results of all samples have good robustness, namely, commercial insurance has positive influence on the health status of residents. After adding a series of control variable, the significance and regression coefficients of each control variable have not changed much. In conclusion, regression results based on all samples are robust.

**Table 4:** Robustness test (1).

**Variable**	**OLS**		**Logit**		**Tobit**	
	**(1)**	**(2)**	**(3)**	**(4)**	**(5)**	**(6)**
Binsurance	0.2061*** (0.0160)	0.0482 *** (0.0160)	0.2924*** (0.0235)	0.0749 *** (0.0252)	0.2061*** (0.0160)	0.0482*** (0.0160)
Age		−0.0524*** (0.0029)		−0.0827*** (0.0047)		−0.0524*** (0.0029)
Age_2		0.0003*** (0.0000)		0.0005*** (0.0000)		0.0003*** (0.0000)
Gender		0.2131*** (0.0144)		0.3463*** (0.0230)		0.2131*** (0.0144)
Edu		0.0491*** (0.0175)		0.0871*** (0.0282)		0.0491*** (0.0175)
Marriage		0.1265*** (0.0290)		0.1931*** (0.0453)		0.1265*** (0.0290)
Household		−0.0245 (0.0181)		−0.0286 (0.0283)		−0.0245 (0.0181)
Ln_fincome		0.0310*** (0.0059)		0.0538*** (0.0096)		0.0310*** (0.0059)
Neighbor		0.0576*** (0.0033)		0.0953*** (0.0055)		0.0576*** (0.0033)
Familysize		0.0058 (0.0037)		0.0077 (0.0059)		0.0058 (0.0037)
Sinsurance		0.0005 (0.0003)		0.0006 (0.0005)		0.0005 (0.0003)
Invest		0.2024*** (0.0674)		0.3241*** (0.1047)		0.2024*** (0.0674)
N	26,105	26,105	26,105	26,105	26,105	26,105
*R* ^2^	0.0063	0.1371	0.0020	0.0498	0.0019	0.0454

The *** in the table respectively show the significance under the significance level of 1%, and the robust standard errors are shown in the brackets.

#### Robustness Test by Time-Phased Regression Test

Self-rated health status of residents fall into variable of ordered discrete type, using numbers from 1 to 5 to show the increasingly better status. Then, the health level is re-divided according to a new classification: 1 to 3 of the self-rated health status of the residents are set to be unhealthy, and 4 to 5 are set to be healthy. According to this new classification, probit regression is conducted on the basis of total samples. As shown in Table 5, the regression result of commercial insurance variable is significantly positive, meaning that commercial insurance can have significant enhancement on health status of urban and rural residents, which shows that the standard regression result has strong robustness.

**Table 5:** Robustness test (2).

**Variable**	**Probit health dichotomy**
Binsurance	0.1140** (0.0518)
Age	−0.0698*** (0.0062)
Age_2	0.0004*** (0.0001)
Gender	0.2125*** (0.0291)
Edu	0.1888*** (0.0382)
Marriage	0.2676*** (0.0664)
Household	0.0671* (0.0347)
Ln_fincome	0.0603*** (0.0089)
Neighbor	−0.0379*** (0.0076)
Familysize	−0.0016 (0.0114)
Sinsurance	−0.0159 (0.0612)
Invest	0.1258** (0.0546)
N	8,675
R^2^	0.1041

The ***, **, and * in the table respectively show the significance under the significance level of 1%, 5%, and 10%, and the robust standard errors are shown in the brackets.

## Further Discussion

### Heterogeneity Test

As can be seen from Table 6, there are 3,679 urban samples and 4,996 rural samples. In the regression results of urban and rural samples and the regression results of commercial insurance, the core explained variable have passed the significant testing and the regression coefficients are positive. This means that, as to urban and rural residents, participating in commercial insurance and health status have a positive correlation. The result of significant testing shows that the data of rural area is better than that of the urban area, and the regression coefficient of rural samples is larger than that of urban samples, meaning that the participation of rural residents in commercial insurance has greater influence on health status than that of urban residents.

**Table 6:** Heterogeneity test–urban-rural differences.

**Variable**	**OLS**		**Logit**		**Tobit**	
	**Rural area**	**Urban area**	**Rural area**	**Urban area**	**Rural area**	**Urban area**
	**(1)**	**(2)**	**(3)**	**(4)**	**(5)**	**(6)**
Binsurance	0.1261** (0.0633)	0.0734* (0.0420)	0.2516** (0.1181)	0.1633* (0.0863)	0.1261** (0.0632)	0.0734* (0.0419)
Age	−0.0523*** (0.0060)	−0.0425*** (0.0061)	−0.1017*** (0.0112)	−0.0973*** (0.0127)	−0.0523*** (0.0060)	−0.0425*** (0.0061)
Age_2	0.0003*** (0.0001)	0.0002*** (0.0001)	0.0006*** (0.0001)	0.0005*** (0.0001)	0.0003*** (0.0001)	0.0002*** (0.0001)
Gender	0.1996*** (0.0287)	0.1304*** (0.0301)	0.3589*** (0.0530)	0.2952*** (0.0620)	0.1996*** (0.0286)	0.1304*** (0.0301)
Edu	0.1900*** (0.0431)	0.1003*** (0.0346)	0.2955*** (0.0801)	0.1842** (0.0712)	0.1900*** (0.0430)	0.1003*** (0.0345)
Marriage	0.2518*** (0.0631)	0.1549** (0.0614)	0.3924*** (0.1176)	0.3199** (0.1264)	0.2518*** (0.0630)	0.1549** (0.0613)
Ln_fincome	0.0788*** (0.0082)	0.0526*** (0.0111)	0.1424*** (0.0157)	0.0910*** (0.0236)	0.0788*** (0.0082)	0.0526*** (0.0110)
Neighbor	−0.0358*** (0.0078)	−0.0277*** (0.0074)	−0.0627*** (0.0146)	−0.0591*** (0.0154)	−0.0358*** (0.0078)	−0.0277*** (0.0074)
Familysize	−0.0040 (0.0104)	−0.0072 (0.0136)	−0.0116 (0.0193)	−0.0034 (0.0283)	−0.0040 (0.0104)	−0.0072 (0.0135)
Sinsurance	−0.0395 (0.0593)	0.0323 (0.0611)	−0.0508 (0.1101)	0.0706 (0.1286)	−0.0395 (0.0592)	0.0323 (0.0610)
Invest	−0.0433 (0.0816)	0.0991** (0.0421)	−0.0828 (0.1505)	0.2318*** (0.0857)	−0.0433 (0.0815)	0.0991** (0.0421)
N	4,996	3,679	4,996	3,679	4,996	3,679
R^2^	0.1752	0.1594	0.0660	0.0655	0.0639	0.0620

The ***, **, and * in the table respectively show the significance under the significance level of 1%, 5%, and 10%, and the robust standard errors are shown in the brackets. The same as in the following tables.

Therefore, it can be concluded that there is a positive correlation between commercial insurance and the health status of urban and rural residents, and there is heterogeneity among rural and urban samples, wherein the correlation is more significant among rural samples.

### Mediation Effect Test

This study adopts the step-test regression coefficient method to study the detailed influence mechanism of commercial insurance purchase on the health status of residents.

In this test, the independent variable is commercial insurance, and the dependent variable is the health status of residents. This study selects household income as a mediating variable. This means, to conduct research on whether participating in commercial insurance can have further effect on the health status of residents by influencing the household income.


(3) $$\begin{array}{lll} Y & = & cX+e_{1} \end{array}$$

(4) $$\begin{array}{lll} M & = & aX+e_{2} \end{array}$$

(5) $$\begin{array}{lll} Y & = & c^{\prime }X+bM+e_{3} \end{array}$$

Step-test regression coefficient is the method most commonly used to test mediation effect. There are three steps. The first step is to test the total effect of the independent variable X on the dependent variable Y, which means to test the coefficient c of equation (3) that which specifically refers to the total effect of participating in commercial insurance on the health status of residents, as shown in Table 7.

**Table 7:** The direct effect.

**Health**	**Coef**	**Std.err**	**t**	***p*** **> |t|**	**[95% Conf. Interval]**	
Binsurance	0.3236191	0.0373225	8.67	0.000	0.2504582	0.3967799
_cons	3.60528	0.011833	304.68	0.000	3.582085	3.628475

As can be seen from Table 7, the coefficient c is significant. This means that there is significant correlation between the health status of residents and participating in commercial insurance. With the regression coefficient at c = 0.324, the second step can be taken.

The second step is to test the relationship between independent X and mediating variable M which is the coefficient a of equation (4). It refers to the relationship between participation in commercial insurance and household income, as shown in Table 8.

**Table 8:** The mediate effect.

**Ln_fincome**	**Coef**	**Std.err**	**t**	***p*** **> |t|**	**[95% Conf. Interval]**	
Binsurance	0.9778213	0.0618904	15.80	0.000	0.8565014	1.099141
_cons	10.24106	0.0196222	521.91	0.000	10.2026	10.27953

It can be concluded that coefficient a is significant. The regression result shows that participating in commercial insurance significantly increases household income, with the coefficient at a = 0.978, the third step can be taken.

In the third step, the logarithm of household income, the mediating variable is added, which means to test coefficients c' and b in equation (5) after controlling mediating variable M, and to analyze the relationship between the health status of residents and participation in commercial insurance, as shown in Table 9.

**Table 9:** Total effect.

**Health**	**Coef**	**Std.err**	**t**	***p*** **> |t|**	**[95% Conf. Interval]**	
Ln_fincome	0.1229989	0.0063396	19.40	0.000	0.1105718	0.1354259
Binsurance	0.2033482	0.0370621	5.49	0.000	0.1306977	0.2759986
_cons	2.345641	0.0659495	35.57	0.000	2.216365	2.474918

It can be seen that coefficients c' and b are both significant, which means that after adding the logarithm of household income, the significant relationship between the health status of residents and participation in commercial insurance has not changed, with the coefficient at c' = 0.203. There is also significant relationship between logarithm of household income and the health status of residents, with the coefficient at b = 0.123. These show that household income has played the mediating role between participation in commercial insurance and the health status of residents.

The complete explanation of result of step-test regression coefficient method is as follows, the total effect of participation in commercial insurance on the health status of residents is 0.324, which is of significant result, and the direct effect of participation in commercial insurance on the health status of residents is 0.203, which is of significant result, but the influence is not obvious. The mediating effect of participation in commercial insurance in improving the health status of residents by increasing household income is 0.120, and it accounted for 37.03% in the total effect.

## Conclusion and Inspiration

This study uses data of CGSS2015 to conduct research on the influence of the commercial insurance participation on the health status of urban and rural residents of China. The result shows that (1) Participation in commercial insurance has a significantly positive influence on the health status of urban and rural residents, (2) The influence of participation in commercial insurance on the health status of residents has obvious differences between urban and rural areas. Specifically, the level of influence on rural residents is higher than that of urban residents, which means that participation in commercial insurance has more significantly positive influence on the health status of rural residents, (3) The self-rated health status of low income group reflects more of the participation of residents in commercial insurance, and has more dependency on commercial insurance, and (4) Participation in commercial insurance can improve the health status of residents by increasing household income, meaning that household income is the mediating variable, producing partial mediating effect.

In view of the above conclusion, the government should further encourage residents to participate in commercial insurance, in order to further improve the health status of urban and rural residents. It is necessary to enhance the publicity of commercial insurance in each area especially in rural places, and provide more vigorous policy or financial support to each commercial insurance company. In brief, it is important to gradually improve the health status of residents so as to achieve the strategic objective of a “Healthy China,” with full consideration of coordination and balance of the knowledge of residents and acceptance of commercial insurance. It is significant to increase the awareness of commercial insurance participation of the residents, especially the low income group, and to conduct necessary promotional activities of commercial insurance purchase among urban and rural residents so as to help them purchase suitable commercial insurance based on their own condition and family situation, and improve the health status of urban and rural residents.

## Data Availability Statement

The original contributions presented in the study are included in the article/supplementary material, further inquiries can be directed to the corresponding author/s.

## Author Contributions

B-CX: conceptualization, methodology, and software. X-JL: visualization, investigation, and writing—reviewing and editing. M-YG: data curation and writing—original draft preparation. All authors contributed to the article and approved the submitted version.

## Funding

This research was partly supported by the National Natural Science Foundation of China (71903105, 71803101).

## Conflict of Interest

The authors declare that the research was conducted in the absence of any commercial or financial relationships that could be construed as a potential conflict of interest.

## Publisher's Note

All claims expressed in this article are solely those of the authors and do not necessarily represent those of their affiliated organizations, or those of the publisher, the editors and the reviewers. Any product that may be evaluated in this article, or claim that may be made by its manufacturer, is not guaranteed or endorsed by the publisher.
